# Correction: The role of artificial photo backgrounds of shelter dogs on pet profile clicking and the perception of sociability

**DOI:** 10.1371/journal.pone.0315522

**Published:** 2024-12-05

**Authors:** Fiona Lamb, Allison Andrukonis, Alexandra Protopopova

The S2 and S3 Files have been uploaded incorrectly. Please view the correct S2 and S3 Files here,

## Supporting information

S2 File (R)

S3 File (CSV)
