# Supplementary material for: The role of artificial photo backgrounds of shelter dogs on pet profile clicking and the perception of sociability
Source: PLoS One. 2021 Dec 16;16(12):e0255551. doi: 10.1371/journal.pone.0255551 (PMC8675723; doi:10.1371/journal.pone.0255551)
Supplement: S1 Table — Sociability scores between dog ID and background type: sample size (N), mean, standard deviation, median, interquartile range (IQR). (DOCX) [file pone.0255551.s001.docx]

**S1.** **Summary of sociability scores**. Sociability scores between dog ID and background type: sample size (N), mean, standard deviation, median, interquartile range (IQR).

| Dog | Background Type | N | Mean | Standard deviation | Median | IQR |
| --- | --- | --- | --- | --- | --- | --- |
| Liberty | Coloured | 170 | 6.92 | 1.93 | 7 | 2 |
|  | Indoor | 171 | 7.15 | 1.80 | 7 | 2 |
|  | Kennel | 168 | 7.11 | 2.05 | 7 | 2 |
|  | Outdoor | 171 | 7.17 | 1.99 | 7 | 3 |
|  | All | 680 | **7.09** | 1.94 | 7 | 2 |
| Phantom | Coloured | 168 | 7.32 | 1.98 | 8 | 3 |
|  | Indoor | 172 | 7.47 | 1.96 | 8 | 3 |
|  | Kennel | 169 | 7.09 | 2.18 | 8 | 3 |
|  | Outdoor | 171 | 7.02 | 1.96 | 7 | 2 |
|  | All | 680 | **7.23** | 2.02 | 8 | 3 |
| Anakin | Coloured | 170 | 7.58 | 1.72 | 8 | 2 |
|  | Indoor | 171 | 7.67 | 1.67 | 8 | 2 |
|  | Kennel | 171 | 7.43 | 1.71 | 8 | 3 |
|  | Outdoor | 168 | 7.55 | 1.69 | 8 | 2 |
|  | All | 680 | **7.56** | 1.70 | 8 | 2 |
| Rogue | Coloured | 171 | 6.20 | 1.86 | 6 | 2 |
|  | Indoor | 170 | 6.48 | 1.98 | 6 | 3 |
|  | Kennel | 171 | 6.44 | 1.93 | 6 | 3 |
|  | Outdoor | 168 | 6.11 | 1.83 | 6 | 2 |
|  | All | 680 | **6.31** | 1.91 | 6 | 3 |
| All | Coloured | 679 | 7.00 | 1.94 | 6 | 2 |
|  | Indoor | 684 | 7.19 | 1.91 | 7 | 3 |
|  | Kennel | 679 | 7.02 | 2.002 | 6 | 2 |
|  | Outdoor | 678 | 6.96 | 1.94 | 6 | 2 |
| Total | All | 2720 | **7.05** | 1.95 | 7 | 2 |
