# Supplementary material for: The role of artificial photo backgrounds of shelter dogs on pet profile clicking and the perception of sociability
Source: PLoS One. 2021 Dec 16;16(12):e0255551. doi: 10.1371/journal.pone.0255551 (PMC8675723; doi:10.1371/journal.pone.0255551)
Supplement: S1 File — (DOCX) [file pone.0255551.s002.docx]

**S2 Virtual Experiment Question**

Start of Block: Demographics

Q1 Select the age range that applies to you.

- 85 or older
- 75 - 84
- 65 - 74
- 55 - 64
- 45 - 54
- 35 - 44
- 25 - 34
- 18 - 24

Q2 What is your gender?

- Male
- Female
- Non-binary/third gender
- Prefer to self-describe
- Prefer not to say

Display This Question:

If What is your gender? = Prefer to self-describe

Q3 Please self-describe your gender.

________________________________________________________________

________________________________________________________________

________________________________________________________________

________________________________________________________________

________________________________________________________________

| 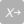 |
| --- |

Q4 In which country do you currently reside?

▼ Afghanistan (1) ... Zimbabwe (1357)

Display This Question:

If List of Countries = United States of America

Q5 In which state do you currently reside?

▼ Alabama (1) ... I do not reside in the United States (53)

Display This Question:

If List of Countries = Canada

Q6 In which province do you currently reside?

▼ British Columbia (1) ... Northwest Territories (13)

Q7 Select all that applied to you.

- I have previously owned a dog(s)
- I currently have a dog(s)
- I have never owned a dog
- I would like to own a dog

Display This Question:

If Select all that applied to you. = I have previously owned a dog(s)

Or Select all that applied to you. = I currently have a dog(s)

| 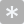 |
| --- |

Q8 Please describe the characteristics (breed, physical traits, personality traits) of your current/previous dog(s).

________________________________________________________________

________________________________________________________________

________________________________________________________________

________________________________________________________________

________________________________________________________________

Display This Question:

If Select all that applied to you. = I would like to own a dog

Q9 Please describe the characteristics (breed, physical traits, personality traits) you are looking for if you were to adopt a dog.

________________________________________________________________

________________________________________________________________

________________________________________________________________

________________________________________________________________

End of Block: Demographics

Start of Block: Instructions

Q10

For the following 4 questions, you will be asked to rank the friendliness/sociability of dogs based on their online photo using a slider. In order to select the score you would like to assign, please click on the grey pointer located at score 0 and drag it to a numerical value. A link below each picture is also provided that will direct you to the dog's adoption page on Petfinder when clicked if you are interested on learning more about the dog.

End of Block: Instructions

Sample Background Question Type: randomized to either present ¼ background types (X4)

Start of Block: Indoor Background

Q11

| 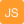 |
| --- |


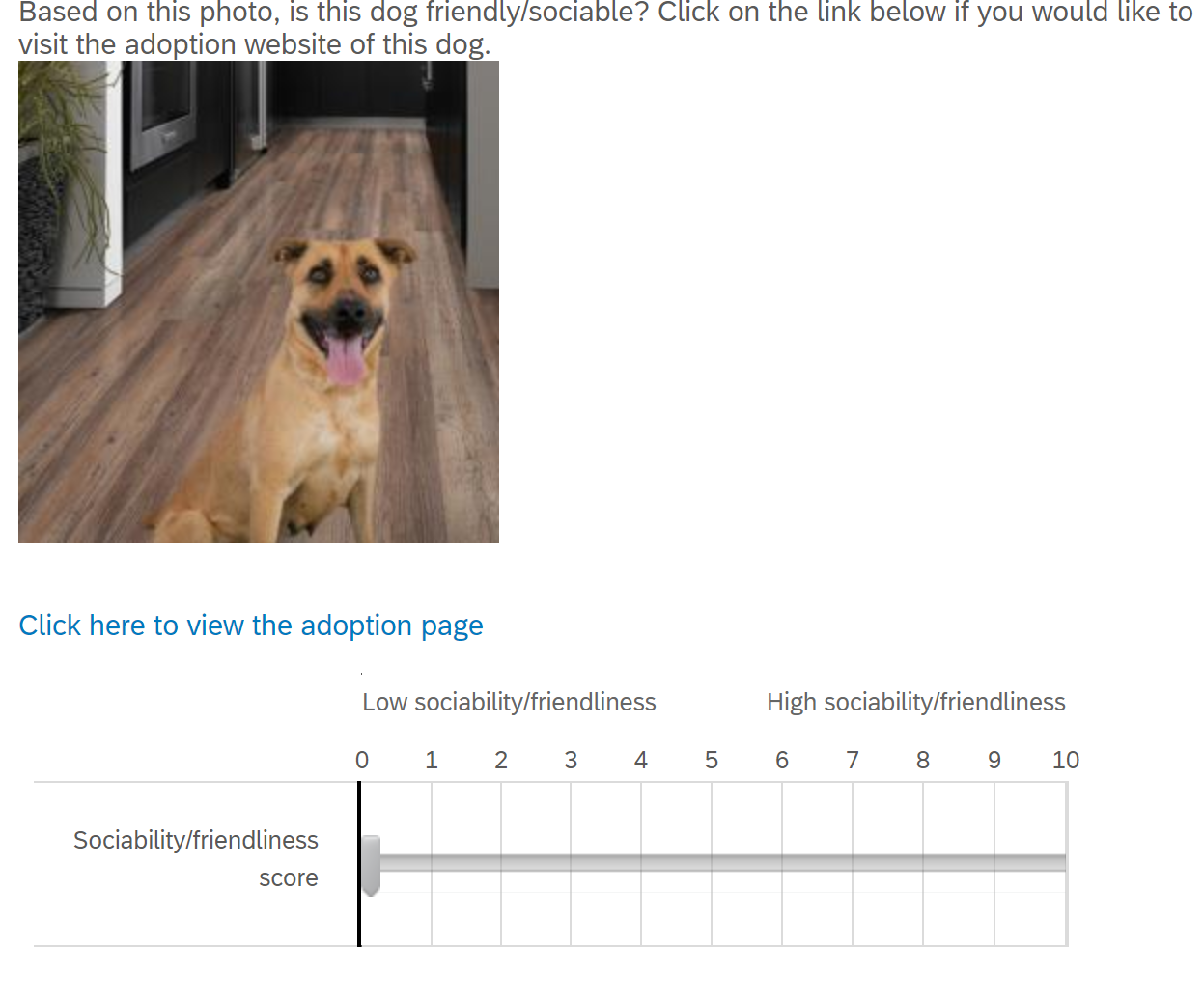


End of Block: Indoor Background

Start of Block: In-Kennel Background

| 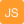 |
| --- |

Q12


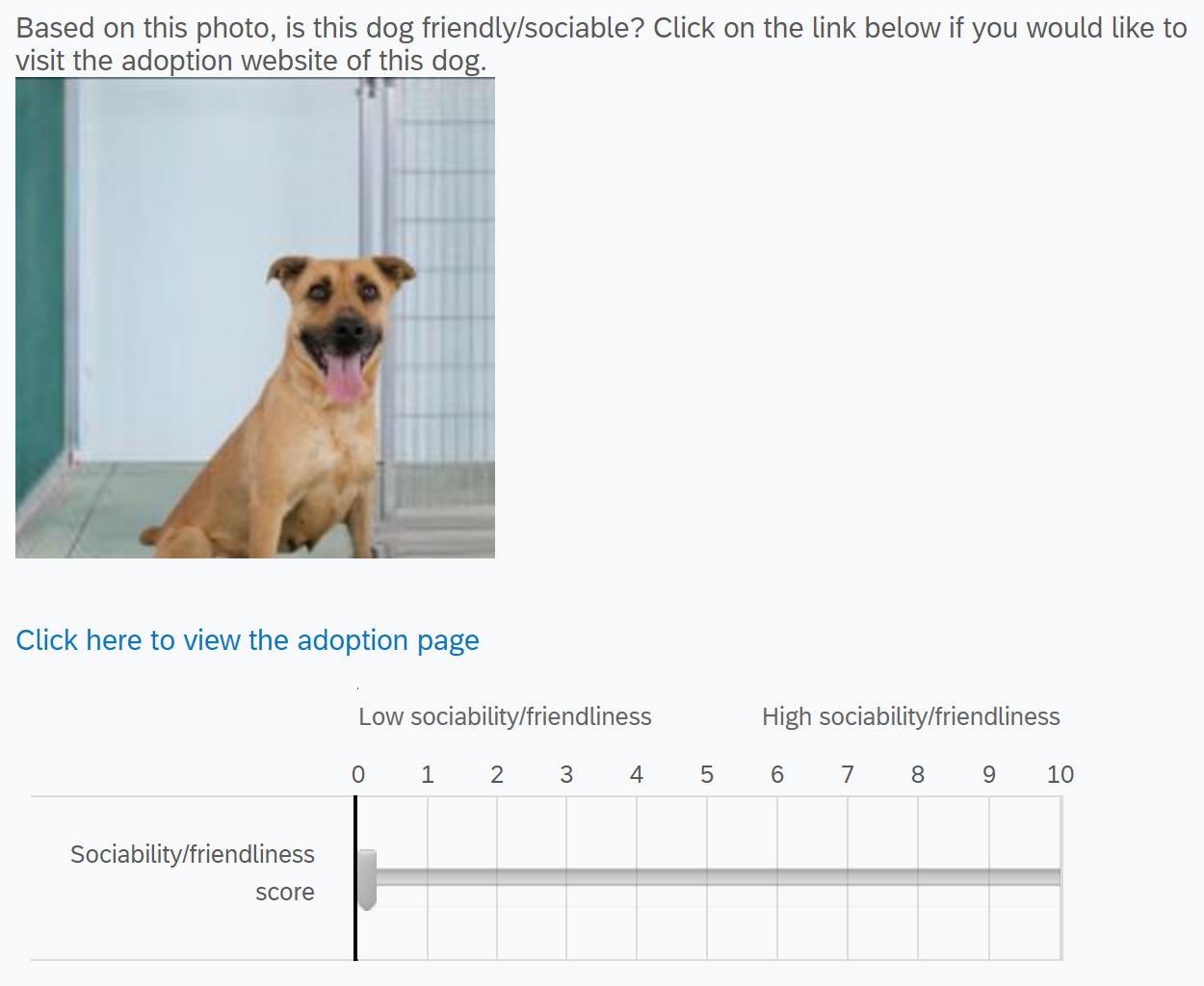


End of Block: In-Kennel Background

Start of Block: Outdoor Background

| 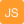 |
| --- |

Q13

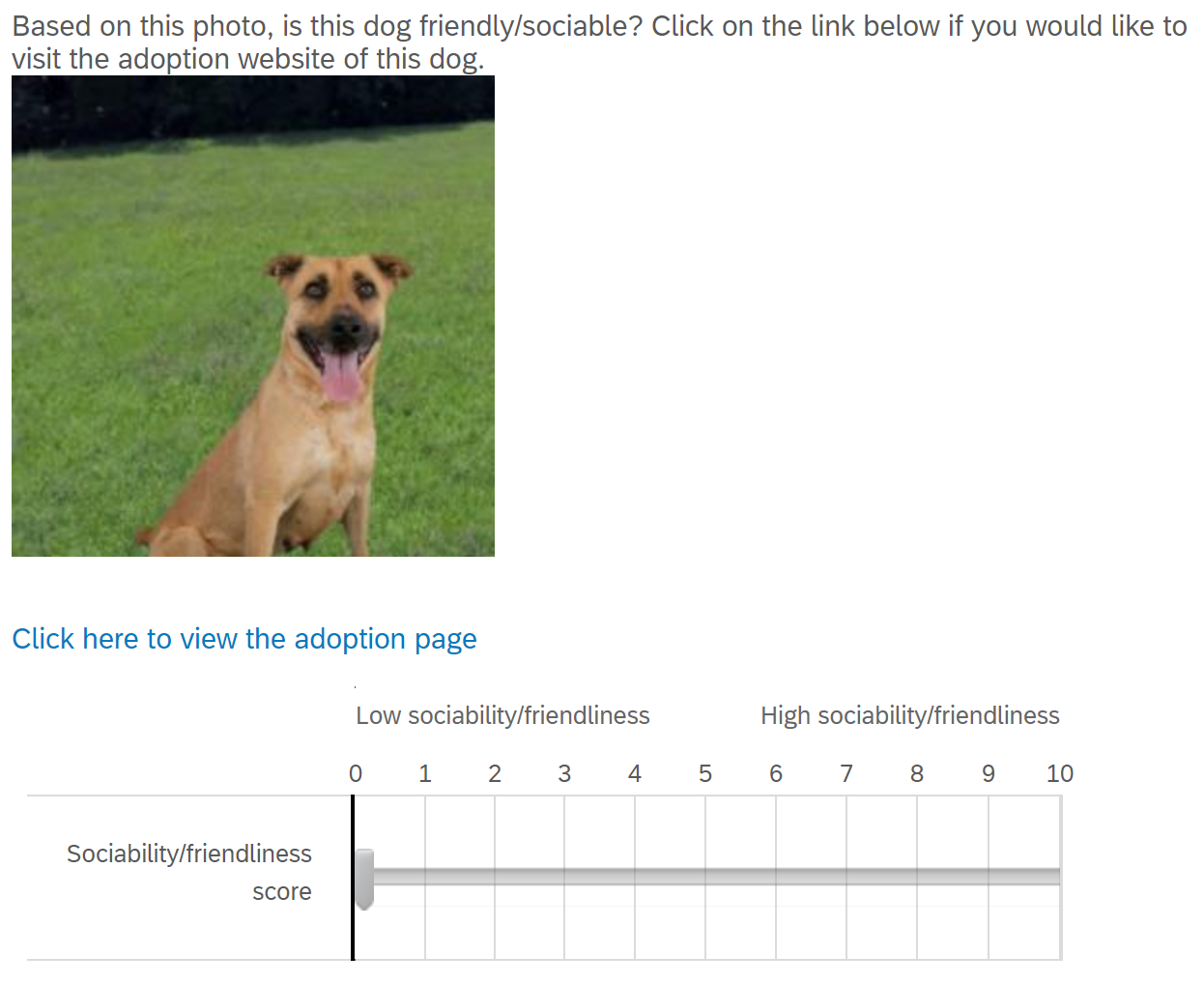


End of Block: Outdoor Background

Start of Block: Colored Background

| 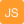 |
| --- |

Q14


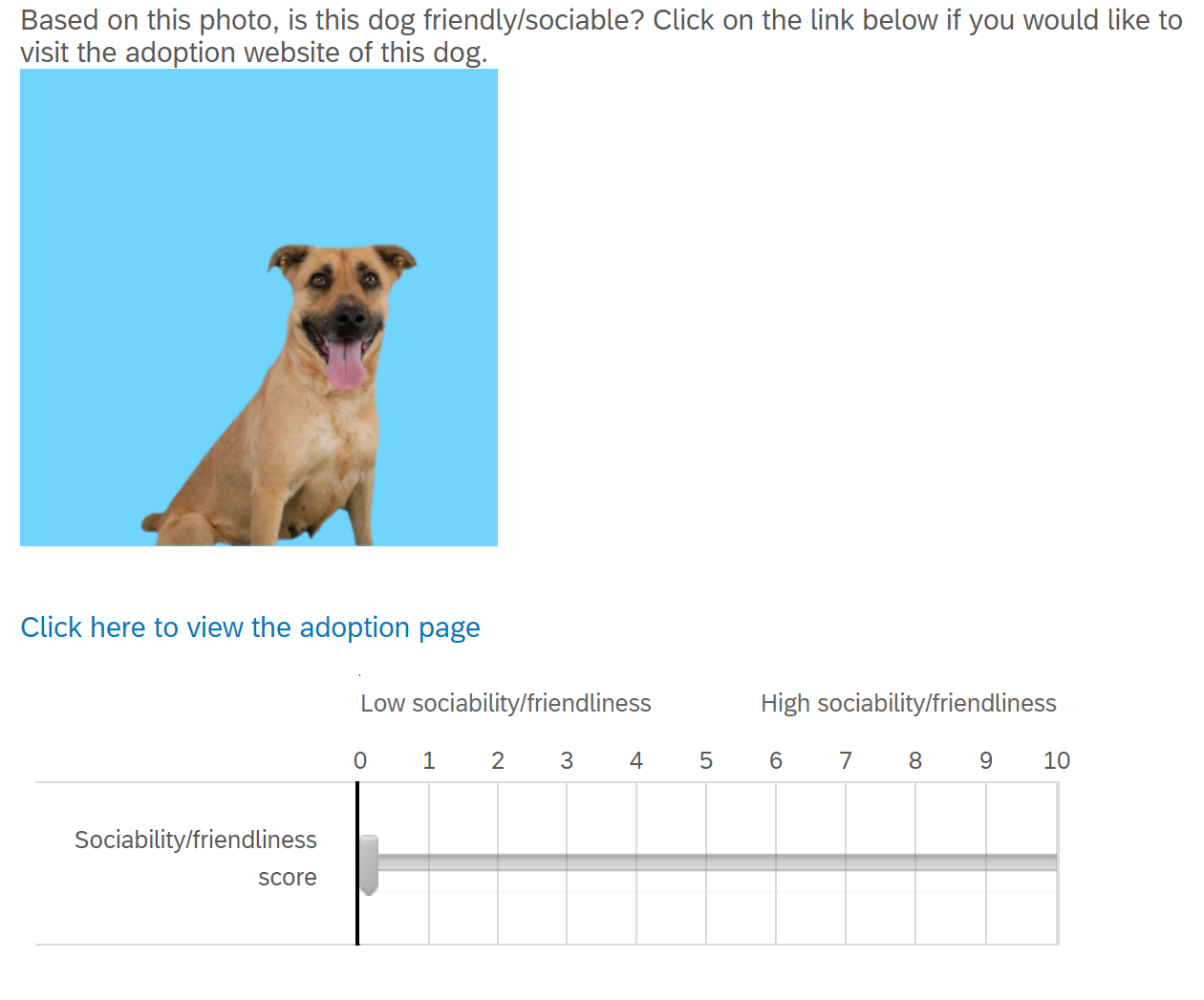


End of Block: Colored Background

Start of Block: Block Feedback 1

Q15
In this survey, participants were initially informed that the purpose of the study is to collect data on the human perception of the personality of dogs based on online photos to help design an algorithm for a software that can recognize positively perceived dogs traits in online photos. However, participants were not informed that the online photo backgrounds of each dog were altered (indoor, in-kennel, outdoor, colored) and randomly displayed. Clicking behavior on the links associated with each dog was also tracked to measure the level of interest in adopting these shelter/foster dogs. This information was withheld to prevent participants' knowledge of the online photo backgrounds from influencing their response to the different photos.

Q16 Were you able to pick up on the differences in photo backgrounds in this survey?

- 1 - Definitely not
- 2 - Probably not
- 3 - Might or might not
- 4 - Probably yes
- 5 - Definitely yes

End of Block: Block Feedback 1
